# Supplementary material for: Cloning and Expression of a Perilla frutescens Cytochrome P450 Enzyme Catalyzing the Hydroxylation of Phenylpropenes
Source: Plants (Basel). 2020 May 1;9(5):577. doi: 10.3390/plants9050577 (PMC7284770; doi:10.3390/plants9050577)
Supplement: Supplementary file 1 [file plants-09-00577-s001.pdf]

Supplementary Data S1. Analytical data for the synthesized compounds.

General

^{1}H and ^{13}C NMR was recorded at 500 and 125 MHz, respectively. Chemical shifts (δ) and coupling constants (*J*) are presented in parts per million relative to tetramethylsilane and hertz, respectively. Abbreviations are as follows: s, singlet; d, doublet; t, triplet; m, multiplet; br, broad. ^{13}C peak multiplicity assignments were made based on DEPT data. The wave numbers of maximum absorption peaks of IR spectroscopy are presented in cm^{-1}.

6-Allyl-2,3-dimethoxyphenol (5)

2-Benzenesulfonyl-1,3-dibromopropane [219500-61-5] (1): ^{1}H NMR (500 MHz, $CDCl_3$): δ 3.57 (tt, *J* = 6.5, 4.5 Hz, 1H), 3.82 (dd, *J* = 11.0, 6.5 Hz, 2H), 3.90 (dd, *J* = 11.0, 4.0 Hz, 2H), 7.62 (t, *J* = 7.5 Hz, 2H), 7.74 (t, *J* = 7.5 Hz, 1H), 7.95 (d, *J* = 7.5 Hz, 2H). ^{13}C NMR (125 MHz, $CDCl_3$): δ 26.2 (CH_2), 65.9 (CH), 129.2 (CH), 129.6 (CH), 134.8 (CH), 137.3 (C).

1-(2-Benzenesulfonylallyloxy)-2,3-dimethoxybenzene (2): ^{1}H NMR (500 MHz, $CDCl_3$): δ 3.74 (s, 3H), 3.84 (s, 3H), 4.77 (s, 2H), 6.20 (s, 1H), 6.30 (d, *J* = 8.5 Hz, 1H), 6.54 (s, 1H), 6.58 (d, *J* = 8.5 Hz, 1H), 6.87 (t, *J* = 8.5 Hz, 1H), 7.57 (t, *J* = 7.5 Hz, 2H), 7.66 (t, *J* = 7.5 Hz, 1H), 7.93 (d, *J* = 7.5 Hz, 2H). ^{13}C NMR (125 MHz, $CDCl_3$): δ 55.9 (CH_3), 60.7 (CH_3), 65.3 (CH_2), 106.2 (CH), 107.0 (CH), 123.6 (CH), 125.7 (CH_2), 128.0 (CH), 129.3 (CH), 133.7 (CH), 138.5 (C), 138.8 (C), 146.1 (C), 151.2 (C), 153.6 (C). IR (neat): 2928, 2855, 1682, 1597, 1467, 1474, 1447, 1319, 1254, 1200, 1142, 1111, 1080, 1007, 991, 910, 837, 775, 737 cm^{-1}. EIMS (*m/z*): 336 (M + 2), 335 (M + 1), 334 (M^+), 193 (M – $PhSO_2$), 192 (M – $PhSO_2H$), 177 (M – $PhSO_2H$ – Me), 153 (M – $CH_2CH(SO_2Ph)CH_2$), 125, 110, 95, 93. HRMS–FAB (*m/z*): $[M + H]^+$ calcd for $C_{17}H_{19}O_5S$, 335.0953; found, 335.0945.

6-(2-Benzenesulfonylallyl)-2,3-dimethoxyphenol (3): ^{1}H NMR (500 MHz, $CDCl_3$): δ 3.52 (s, 2H), 3.82 (s, 3H), 3.83 (s, 3H), 5.50 (s, 1H), 5.70 (s, 1H), 6.36 (d, *J* = 8.5 Hz, 1H), 6.38 (s, 1H), 6.66 (d, *J* = 8.5 Hz, 1H), 7.52 (t, *J* = 7.5 Hz, 2H), 7.61 (t, *J* = 7.5 Hz, 1H), 7.89 (d, *J* = 7.5 Hz, 2H). ^{13}C NMR (125 MHz, $CDCl_3$): δ 29.7 (CH_2), 55.7 (CH_3), 60.9 (CH_3), 103.5 (CH), 115.0 (C), 124.6 (CH_2), 125.3 (CH), 128.1 (CH), 128.9 (CH), 133.2 (CH), 135.3 (C), 139.0 (C), 147.4 (C), 149.1 (C), 151.4 (C). IR (neat): 3426, 3001, 2940, 2909, 2839, 1616, 1470, 1447, 1431, 1304, 1242, 1219, 1204, 1161, 1130, 1096, 1080, 1038, 972, 953, 910, 745 cm^{-1}. EIMS (*m/z*): 336 (M + 2), 335 (M + 1), 334 (M^+), 192 (M – $PhSO_2H$), 177 (M – $PhSO_2H$ – Me). HRMS–FAB (*m/z*): $[M + H]^+$ calcd for $C_{17}H_{19}O_5S$, 335.0953; found, 335.0954.

3-Benzenesulfonyl-7,8-dimethoxychromane (4): ^{1}H NMR (500 MHz, $CDCl_3$): δ 3.02 (dd, *J* = 16.0,

5.0 Hz, 1H), 3.19 (dd, *J* = 16.0, 11.0 Hz, 1H), 3.60 (dddd, *J* = 11.0, 10.5, 5.0, 3.0 Hz, 1H), 3.78 (s, 3H), 3.81 (s, 3H), 4.12 (t, *J* = 10.5 Hz, 1H), 4.59 (dd, *J* = 10.5, 3.0 Hz, 1H), 6.19 (d, *J* = 8.5 Hz, 1H), 6.73 (d, *J* = 8.5 Hz, 1H), 7.59 (dd, *J* = 8.0, 7.0 Hz, 2H), 7.69 (t, *J* = 7.0 Hz, 1H), 7.93 (d, *J* = 8.0 Hz, 2H). ^{13}C NMR (125 MHz, $CDCl_3$): δ 24.5 (CH_2), 56.1 (CH_3), 57.6 (CH), 60.9 (CH_3), 63.9 (CH_2), 105.5 (CH), 112.3 (C), 123.7 (CH), 128.7 (CH), 129.4 (CH), 134.2 (CH), 137.1 (C), 137.2 (C), 147.1 (C), 152.0 (C). IR (neat): 3017, 2940, 2836, 1612, 1582, 1501, 1462, 1447, 1308, 1288, 1227, 1200, 1150, 1111, 1072, 1049, 968, 791, 756 cm^{-1}. EIMS (*m*/*z*): 336 (M + 2), 335 (M + 1), 334 (M^+), 192 (M – $PhSO_2H$), 177 (M – $PhSO_2H$ – Me). HRMS–FAB (*m*/*z*): $[M + H]^+$ calcd for $C_{17}H_{19}O_5S$, 335.0953; found, 335.0945.

6-Allyl-2,3-dimethoxyphenol [450357-58-1] (5): ^{1}H NMR (500 MHz, $CDCl_3$): δ 3.35 (d, *J* = 6.5 Hz, 2H), 3.85 (s, 3H), 3.90 (s, 3H), 5.04 (d, *J* = 10.0 Hz, 1H), 5.09 (d, *J* = 17.0 Hz, 1H), 5.86 (s, 1H), 5.99 (ddt, *J* = 17.0, 10.0, 6.5 Hz, 1H), 6.43 (d, *J* = 8.5 Hz, 1H), 6.79 (d, *J* = 8.5 Hz, 2H). ^{13}C NMR (125 MHz, $CDCl_3$): δ 33.5 (CH_2), 55.7 (CH_3), 60.8 (CH_3), 103.4 (CH), 115.2 (CH_2), 119.1 (C), 124.1 (CH), 135.3 (C), 136.9 (CH), 147.2 (C), 150.7 (C). IR (neat): 3507, 3078, 3001, 2974, 2939, 2835, 2909, 1639, 1616, 1589, 1504, 1466, 1431, 1315, 1277, 1219, 1161, 1096, 1038, 995, 972, 910, 791, 737 cm^{-1}. EIMS (*m*/*z*): 194 (M^+), 179 (M – CH_3), 163 (M – OCH_3), 147, 119, 91.

1-(Allyloxy)-2,3-dimethoxybenzene [380621-78-3] (6): ^{1}H NMR (500 MHz, $CDCl_3$): δ 3.86 (s, 3H), 3.87 (s, 3H), 4.60 (d, *J* = 5.0 Hz, 2H), 5.27 (dd, *J* = 10.5, 1.5 Hz, 1H), 5.41 (dd, *J* = 17.0, 1.5 Hz, 1H), 6.07 (ddt, *J* = 17.0, 10.5, 5.0 Hz, 1H), 6.58 (d, *J* = 8.5 Hz, 2H), 6.96 (t, *J* = 8.5 Hz, 1H). ^{13}C NMR (125 MHz, $CDCl_3$): δ 56.0 (CH_3), 60.7 (CH_3), 69.8 (CH_2), 105.4 (CH), 107.1 (CH), 117.4 (CH_2), 123.4 (CH), 133.4 (CH), 138.6 (C), 152.4 (C), 153.6 (C). IR (neat): 3086, 2997, 2936, 2889, 2832, 1593, 1493, 1474, 1300, 1258, 1177, 1103, 1007, 914, 775, 733 cm^{-1}. EIMS (*m*/*z*): 194 (M^+), 179 (M – Me), 153, 125, 110, 95, 91. ^{1}H NMR is in consistent with that reported [1].

4-Allyl-2,3-dimethoxyphenol [29445-64-5] (7): ^{1}H NMR (500 MHz, $CDCl_3$): δ 3.32 (d, *J* = 6.5 Hz, 2H), 3.83 (s, 3H), 3.92 (s, 3H), 5.03 (d, *J* = 16.5 Hz, 1H), 5.06 (d, *J* = 10.0 Hz, 1H), 5.63 (s, 1H), 5.95 (ddt, *J* = 16.5, 10.0, 6.5 Hz, 1H), 6.67 (d, *J* = 8.5 Hz, 1H), 6.78 (d, *J* = 8.5 Hz, 1H). ^{13}C NMR (125 MHz, $CDCl_3$): δ 33.6 (CH_2), 60.5 (CH_3), 60.7 (CH_3), 110.2 (CH), 115.2 (CH_2), 124.7 (CH), 125.2 (C), 137.7 (CH), 139.7 (C), 148.0 (C), 150.7 (C).

2-Allyl-4,5-dimethoxyphenol (8)

2-Allyl-4,5-dimethoxyphenol [59893-87-7] (8): ^{1}H NMR (500 MHz, $CDCl_3$): δ 3.35 (dt, *J* = 6.5, 1.5 Hz, 2H), 3.83 (s, 3H), 3.84 (s, 3H), 4.72 (s, 1H), 5.14–5.20 (m, 2H), 6.00 (ddt, *J* = 17.5, 9.5, 6.5 Hz, 1H), 6.47 (s, 1H), 6.62 (s, 1H). ^{13}C NMR (125 MHz, $CDCl_3$): δ 34.6 (CH_2), 55.8 (CH_3), 56.5 (CH_3), 101.2 (CH), 113.8 (CH), 115.9 (C), 116.1 (CH_2), 136.6 (CH), 142.8 (C), 148.0 (C), 148.3 (C). IR

(neat): 3445, 3078, 3001, 2936, 2913, 2835, 1639, 1616, 1520, 1450, 1412, 1292, 1238, 1200, 1111, 1030, 995, 914, 845, 756, 733 cm^{-1}. EIMS (*m*/*z*): 194 (M^+), 179 (M – Me), 123, 91.

2-Allyl-3,4-dimethoxyphenol [66967-26-8] (9): ^{1}H NMR (500 MHz, $CDCl_3$): δ 3.48 (d, *J* = 6.0 Hz, 2H), 3.80 (s, 3H), 3.81 (s, 3H), 4.97 (br s, 1H), 5.08–5.14 (m, 2H), 6.01 (ddt, *J* = 17.5, 10.0, 6.0 Hz, 1H), 6.56 (d, *J* = 9.0 Hz, 1H), 6.70 (d, *J* = 9.0 Hz, 1H). ^{13}C NMR (125 MHz, $CDCl_3$): δ 28.3 (CH_2), 56.3 (CH_3), 61.0 (CH_3), 110.5 (CH), 111.2 (CH), 115.7 (CH_2), 120.3 (C), 136.3 (CH), 147.0 (C), 147.7 (C), 148.7 (C). IR (neat): 3387, 3074, 3001, 2940, 2832, 1697, 1639, 1601, 1493, 1470, 1427, 1339, 1254, 1215, 1177, 1111, 1080, 1042, 995, 968, 910, 853, 799, 764, 737 cm^{-1}. EIMS (*m*/*z*): 194 (M^+), 179 (M – Me), 147, 119, 91.

5-Allyl-2,3-dimethoxyphenol (14)

3-*tert*-Butyldimethylsiloxy-4,5-dimethoxybenzenemethanol [111394-55-9] (11): ^{1}H NMR (500 MHz, $CDCl_3$): δ 0.18 (s, 6H), 1.01 (s, 9H), 1.57 (t, *J* = 6.0 Hz, 1H), 3.77 (s, 3H), 3.86 (s, 3H), 4.58 (d, *J* = 6.0 Hz, 2H), 6.50 (d, *J* = 2.0 Hz, 1H), 6.59 (d, *J* = 2.0 Hz, 1H). IR (neat): 3426, 2951, 2932, 2859, 1585, 1501, 1454, 1427, 1342, 1231, 1115, 1003, 837 cm^{-1}. The IR was in good agreement with that reported.

3-*tert*-Butyldimethylsiloxy-4,5-dimethoxybenzyl Bromide [111394-56-0] (12): ^{1}H NMR (500 MHz, $CDCl_3$): δ 0.18 (s, 6H), 1.00 (s, 9H), 3.78 (s, 3H), 3.86 (s, 3H), 4.41 (s, 2H), 6.54 (d, *J* = 2.0 Hz, 1H), 6.58 (d, *J* = 2.0 Hz, 1H). ^{13}C NMR (125 MHz, $CDCl_3$): δ –4.8 (CH_3), 18.1 (C), 25.5 (CH_3), 33.9 (CH_2), 55.8 (CH_3), 60.2 (CH_3), 106.1 (CH), 114.8 (CH), 132.8 (C), 140.4 (C), 149.2 (C), 153.6 (C). IR (neat): 2955, 2932, 2889, 2859, 1585, 1501, 1454, 1427, 1346, 1234, 1211, 1126, 1111, 1007, 910, 837, 783, 737 cm^{-1}. EIMS (*m*/*z*): 362 (M+2), 360 (M^+), 347 (M+2 – Me), 345 (M – Me), 305 (M+2 – *t*-Bu), 303 (M – *t*-Bu), 281 (M – Br), 209.

5-Allyl-1-*tert*-butyldimethylsiloxy-2,3-dimethoxybenzene (13): ^{1}H NMR (500 MHz, $CDCl_3$): δ 0.17 (s, 6H), 1.00 (s, 9H), 3.28 (d, *J* = 7.0 Hz, 2H), 3.76 (s, 3H), 3.83 (s, 3H), 5.05–5.11 (m, 2H), 5.94 (ddt, *J* = 17.0, 10.0, 7.0 Hz, 1H), 6.34 (d, *J* = 2.0 Hz, 1H), 6.37 (d, *J* = 2.0 Hz, 1H). ^{13}C NMR (125 MHz, $CDCl_3$): δ –4.7 (CH_3), 18.3 (C), 25.7 (CH_3), 40.1 (CH_2), 55.8 (CH_3), 60.3 (CH_3), 105.6 (CH), 114.1 (CH), 115.7 (CH_2), 135.4 (C), 137.3 (CH), 138.5 (C), 149.2 (C), 153.5 (C). IR (neat): 2955, 2932, 2897, 2859, 1585, 1501, 1454, 1427, 1342, 1234, 1115, 1011, 910, 887, 783 cm^{-1}. EIMS (*m*/*z*): 308 (M^+), 251 (M – *t*-Bu), 236 (M – *t*-Bu – Me). HRMS–FAB (*m*/*z*): $[M + H]^+$ calcd for $C_{17}H_{29}O_3Si$, 309.1886; found, 309.1888.

5-Allyl-2,3-dimethoxyphenol [76773-99-4] (14): ^{1}H NMR (500 MHz, $CDCl_3$): δ 3.29 (d, *J* = 7.0 Hz,

2H), 3.85 (s, 3H), 3.87 (s, 3H), 5.07 (d, *J* = 10 Hz, 1H), 5.10 (d, *J* = 17.0 Hz, 1H), 5.70 (s, 1H), 5.94 (ddt, *J* = 17.0, 10.0, 7.0 Hz, 1H), 6.30 (s, 1H), 6.45 (s, 1H). ^{13}C NMR (125 MHz, $CDCl_3$): δ 40.2 (CH_2), 55.7 (CH_3), 60.9 (CH_3), 104.3 (CH), 108.1 (CH), 115.9 (CH_2), 133.7 (C), 136.3 (C), 137.1 (CH), 149.1 (C), 152.2 (C). IR (neat): 3456, 3075, 3005, 2936, 2909, 2839, 1593, 1508, 1462, 1431, 1350, 1234, 1200, 1065, 1138, 1107, 995, 914, 821, 775 cm^{-1}. EIMS (*m*/*z*): 194 (M^+), 179 (M – Me), 11 9, 91. ^1H and ^{13}C NMR were identical to those reported [2].

6-Allyl-4-methoxy-1,3-benzodioxol-5-ol (20)

4-Methoxy-1,3-benzodioxole [1817-95-4] (15): ^1H NMR (500 MHz, $CDCl_3$): δ 3.90 (s, 3H), 5.96 (s, 2H), 6.53 (d, *J* = 8.0 Hz, 2H), 6.79 (t, *J* = 8.0 Hz, 1H). ^{13}C NMR (125 MHz, $CDCl_3$): δ 56.2 (CH_3), 100.9 (CH_2), 102.1 (CH), 107.2 (CH), 121.8 (CH), 135.0 (C), 143.9 (C), 148.5 (C). IR (neat): 2955, 2940, 2920, 2905, 2835, 1636, 1504, 1462, 1350, 1285, 1254, 1180, 1088, 1034, 961, 926, 833, 756, 710 cm^{-1}. EIMS (*m*/*z*): 152 (M^+), 151 (M – H), 137 (M – CH_3), 107. The mp, ^1H NMR, and IR are in good agreement with those reported [3,4,5].

4-Methoxy-1,3-benzodioxole-5-carbaldehyde [5779-99-7] (16): ^1H NMR (500 MHz, $CDCl_3$): δ 4.13 (s, 3H), 6.04 (s, 2H), 6.60 (d, *J* = 8.5 Hz, 1H), 7.47 (d, *J* = 8.5 Hz, 1H), 10.23 (s, 1H). ^{13}C NMR (125 MHz, $CDCl_3$): δ 60.0 (CH_3), 101.8 (CH_2), 103.1 (CH), 122.6 (C), 124.1 (CH), 135.8 (C), 146.0 (C), 154.6 (C), 188.0 (CH). IR (neat): 3001, 2943, 2916, 2859, 2789, 1663, 1612, 1597, 1470, 1408, 1346, 1277, 1242, 1223, 1076, 1038, 926, 806, 787 cm^{-1}. EIMS (*m*/*z*): 180 (M^+), 164, 151 (M – CHO). The mp, ^1H NMR, and IR are in good agreement with those reported [6,7].

7-Methoxy-1,3-benzodioxole-4-carbaldehyde [23731-55-7] (17): ^1H NMR (500 MHz, $CDCl_3$): δ 3.99 (s, 3H), 6.15 (s, 2H), 6.63 (d, *J* = 9.0 Hz, 1H), 7.31 (d, *J* = 9.0 Hz, 1H), 9.99 (s, 1H). ^{13}C NMR (125 MHz, $CDCl_3$): δ 56.7 (CH_3), 102.8 (CH_2), 107.7 (CH), 114.3 (C), 123.5 (CH), 135.5 (C), 148.2 (C), 150.2 (C), 186.9 (C). IR (neat): 3093, 3021, 2978, 2913, 2843, 2727, 1686, 1632, 1504, 1447, 1400, 1292, 1261, 1231, 1207, 1173, 1111, 1099, 1026, 957, 914, 787, 764, 737 cm^{-1}. EIMS (*m*/*z*): 180 (M^+), 151 (M – CHO). The mp, ^1H NMR, and IR are in good agreement with those reported [4,5].

4-Methoxy-1,3-benzodioxol-5-ol [23504-78-1] (18): ^1H NMR (500 MHz, $CDCl_3$): δ 4.05 (s, 3H), 5.31 (s, 1H), 5.89 (s, 2H), 6.38 (d, *J* = 8.0 Hz, 1H), 6.41 (d, *J* = 8.0 Hz, 1H). ^{13}C NMR (125 MHz, $CDCl_3$): δ 59.9 (CH_3), 101.1 (CH_2), 101.8 (CH), 106.0 (CH), 131.4 (C), 136.2 (C), 142.1 (C), 142.7 (C). IR (neat): 3445, 2986, 2947, 2889, 2846, 2778, 1643, 1620, 1493, 1466, 1447, 1404, 1269, 1238, 1200, 1169, 1042, 988, 964, 918, 791, 737 cm^{-1}. EIMS (*m*/*z*): 168 (M^+), 167 (M – H), 153 (M – Me), 123, 121, 199, 97, 95.

5-Allyloxy-4-methoxy-1,3-benzodioxole [23731-59-1] (19): ^{1}H NMR (500 MHz, $CDCl_3$): δ 4.01 (s, 3H), 4.51 (d, *J* = 5.5 Hz, 2H), 5.26 (dd, *J* = 10.5, 1.5 Hz, 1H), 5.38 (dd, *J* = 17.5, 1.5 Hz, 1H), 5.91 (s, 2H), 6.06 (ddt, *J* = 17.5, 10.5, 5.5 Hz, 1H), 6.36 (d, *J* = 8.5 Hz, 1H), 6.42 (d, *J* = 8.5 Hz, 1H). ^{13}C NMR (125 MHz, $CDCl_3$): δ 60.2 (CH_3), 71.1 (CH_2), 101.1 (CH), 101.2 (CH_2), 107.0 (CH), 117.5 (CH_2), 133.5 (CH), 134.8 (C), 138.0 (C), 143.3 (C), 146.1 (C). IR (neat): 3078, 2997, 2940, 2886, 2778, 1632, 1493, 1462, 1234, 1065, 926, 779, 737 cm^{-1}. EIMS (*m*/*z*): 208 (M^+), 167 (M – C_3H_5), 137, 111, 109, 107, 94.

6-Allyl-4-methoxy-1,3-benzodioxol-5-ol [23731-60-4] (20): ^{1}H NMR (500 MHz, $CDCl_3$): δ 3.31 (d, *J* = 6.5 Hz, 2H), 4.04 (s, 3H), 5.04 (d, *J* = 10.0 Hz, 1H), 5.06 (d, *J* = 17.0 Hz, 1H), 5.40 (s, 1H), 5.85 (s, 2H), 5.95 (ddt, *J* = 17.0, 10.0, 6.5 Hz, 1H), 6.33 (s, 1H). ^{13}C NMR (125 MHz, $CDCl_3$): δ 33.7 (CH_2), 59.9 (CH_3), 100.9 (CH_2), 102.8 (CH), 115.3 (CH_2), 117.5 (C), 131.1 (C), 134.2 (C), 136.7 (CH), 139.8 (C), 141.6 (C). IR (neat): 3499, 3075, 3005, 2978, 2947, 2886, 2873, 2774, 1636, 1485, 1466, 1435, 1416, 1285, 1246, 1200, 1177, 1072, 1049, 984, 914, 837, 775, 733 cm^{-1}. EIMS (*m*/*z*): 208 (M^+), 193 (M – Me), 181 (M – C_2H_3), 177 (M – OMe), 163. HRMS–FAB (*m*/*z*): $[M]^+$ calcd for $C_{11}H_{12}O_4$, 208.0736; found, 208.0731.

5-Allyl-7-methoxy-1,3-benzodioxol-4-ol (23)

7-Methoxy-1,3-benzodioxol-4-ol [23812-54-6] (21): ^{1}H NMR (500 MHz, $CDCl_3$): δ 3.85 (s, 3H), 4.58 (s, 1H), 5.99 (s, 2H), 6.41 (d, *J* = 9.0 Hz, 1H), 6.43 (d, *J* = 9.0 Hz, 1H). ^{13}C NMR (125 MHz, $CDCl_3$): δ 57.0 (CH_3), 101.7 (CH_2), 107.9 (CH), 110.1 (CH), 134.2 (C), 135.4 (C), 136.3 (C), 138.3 (C). IR (neat): 3291, 3082, 3051, 3009, 2963, 2909, 2839, 1616, 1520, 1504, 1470, 1420, 1400, 1342, 1246, 1177, 1165, 1088, 1053, 1015, 961, 918, 791, 779, 733 cm^{-1}. EIMS (*m*/*z*): 168 (M^+), 153 (M – Me), 123, 97, 95. The mp and ^{1}H NMR are in agreement with those reported [8].

4-Allyloxy-7-methoxy-1,3-benzodioxole [23731-70-6] (22): ^{1}H NMR (500 MHz, $CDCl_3$): δ 3.86 (s, 3H), 4.58 (d, *J* = 5.5 Hz, 2H), 5.27 (dd, *J* = 10.5, 1.5 Hz, 1H), 5.39 (dd, *J* = 17.0, 1.5 Hz, 1H), 5.99 (s, 2H), 6.05 (ddt, *J* = 17.0, 10.5, 5.5 Hz, 1H), 6.42 (d, *J* = 9.0 Hz, 1H), 6.46 (d, *J* = 9.0 Hz, 1H). ^{13}C NMR (125 MHz, $CDCl_3$): δ 56.7 (CH_3), 70.8 (CH_2), 101.6 (CH_2), 107.0 (CH), 109.2 (CH), 117.8 (CH_2), 133.3 (CH), 136.5 (C), 137.1 (C), 137.5 (C), 139.0 (C). IR (neat): 3082, 3009, 2955, 2936, 2889, 2839, 2778, 1647, 1609, 1512, 1462, 1346, 1265, 1173, 1096, 1076, 1060, 1030, 976, 961, 922, 775, 737 cm^{-1}. EIMS (*m*/*z*): 208 (M^+), 167 (M – C_3H_5), 137, 111, 109, 107, 94.

5-Allyl-7-methoxy-1,3-benzodioxol-4-ol [76773-99-4] (23): ^{1}H NMR (500 MHz, $CDCl_3$): δ 3.34 (d, *J* = 6.5 Hz, 2H), 3.85 (s, 3H), 4.53 (s, 1H), 5.11 (dd, *J* = 10.0, 1.5 Hz, 1H), 5.13 (dd, *J* = 17.0, 1.5 Hz, 1H), 5.97 (s, 2H), 5.98 (ddt, *J* = 17.0, 10.0, 6.5 Hz, 1H), 6.29 (s, 1H). ^{13}C NMR (125 MHz, $CDCl_3$): δ

34.2 (CH_2), 57.0 (CH_3), 101.8 (CH_2), 108.7 (CH), 115.9 (CH_2), 121.0 (C), 132.4 (C), 134.7 (C), 135.8 (C), 136.6 (CH), 137.6 (C). IR (neat): 3337, 3075, 3005, 2978, 2947, 2886, 2873, 2774, 1628, 1516, 1454, 1350, 1231, 1200, 1123, 1042, 953, 914, 822, 741, 710 cm^{-1}. EIMS (*m*/*z*): 208 (M^+), 193 (M – Me), 181 (M – C_2H_3), 177 (M – OMe), 163, 147, 135, 119, 107, 91. HRMS–FAB (*m*/*z*): $[M]^+$ calcd for $C_{11}H_{12}O_4$, 208.0736; found, 208.0734.

5-Allyl-6,7-dimethoxy-1,3-benzodioxol-4-ol (30)

6,7-Dimethoxy-1,3-benzodioxol-4-ol [22934-71-0] (24): ^{1}H NMR (500 MHz, $CDCl_3$): δ 3.79 (s, 3H), 3.91 (s, 3H), 4.57 (s, 1H), 5.92 (s, 2H), 6.08 (s, 1H). ^{13}C NMR (125 MHz, $CDCl_3$): δ 56.7 (CH_3), 60.7 (CH_3), 95.1 (CH), 101.6 (CH_2), 127.9 (C), 128.7 (C), 133.6 (C), 139.3 (C), 147.7 (C). IR (neat): 3375, 2940, 2909, 2889, 2839, 1709, 1651, 1636, 1512, 1466, 1234, 1092, 941, 910, 737 cm^{-1}. EIMS (*m*/*z*): 198 (M^+), 183 (M – Me), 125, 121, 119, 95. The mp is in good agreement with those reported [9].

4-Hydroxy-6,7-dimethoxy-1,3-benzodioxol-5-carbadehyde (25): ^{1}H NMR (500 MHz, $CDCl_3$): δ 3.92 (s, 3H), 3.98 (s, 3H), 6.07 (s, 2H), 10.1 (s, 1H), 11.5 (s, 1H). ^{13}C NMR (125 MHz, $CDCl_3$): δ 60.9, 62.8, 103.0, 110.3, 130.1, 130.3, 141.2, 147.9, 152.1, 193.6. IR (neat): 1667, 1624, 1604, 1489, 1469, 1427, 1400, 1292, 1091, 1049, 959 cm^{-1}. EIMS (*m*/*z*): 226 (M^+), 211 (M – Me), 183, 169, 147, 118. HRMS–ESI (*m*/*z*): $[M + Na]^+$ calcd for $C_{10}H_{10}NaO_6$, 249.0370; found, 249.0368.

4-*tert*-Butyldimethylsilyloxy-6,7-dimethoxy-1,3-benzodioxole-5-methanol (27): ^{1}H NMR (500 MHz, $CDCl_3$): δ 0.20 (s, 6H), 0.99 (s, 9H), 3.83 (s, 3H), 3.92 (s, 3H), 4.62 (s, 2H), 5.87 (s, 2H). ^{13}C NMR (125 MHz, $CDCl_3$): δ –4.5, 18.3, 25.7, 55.6, 60.4, 61.9, 101.1, 119.8, 132.0, 132.2, 134.0, 138.7, 145.8. IR (neat): 3522, 3464, 2931, 2885, 2858, 1616, 1477, 1431, 1257, 1083, 1053 cm^{-1}. EIMS (*m*/*z*): 342 (M^+), 285 (M – *t*-Bu), 270, 255.

5-Allyl-4-*tert*-butyldimethylsilyloxy-6,7-dimethoxy-1,3-benzodioxole (29): ^{1}H NMR (500 MHz, $CDCl_3$): δ 0.19 (s, 6H), 0.99 (s, 9H), 3.30–3.37 (m, 2H), 3.77 (s, 3H), 3.93 (s, 3H), 4.90–5.00 (m, 2H), 5.86 (s, 2H), 5.89–5.98 (m, 1H). ^{13}C NMR (125 MHz, $CDCl_3$): δ –4.2, 18.4, 25.8, 28.4, 60.4, 61.4, 100.8, 114.3, 118.8, 131.9, 132.3, 133.8, 137.1, 137.3, 145.3. IR (neat): 3016, 2931, 2886, 1477, 1435, 1254, 1215, 1053, 910 cm^{-1}. EIMS (*m*/*z*): 352 (M^+), 280 (M – Me – *t*-Bu). HRMS–ESI (*m*/*z*): $[M + H]^+$ calcd for $C_{18}H_{29}O_5Si$, 353.1779; found, 353.1779.

5-Allyl-6,7-dimethoxy-1,3-benzodioxol-4-ol (30): ^{1}H NMR (500 MHz, $CDCl_3$): δ 3.40 (ddd, *J* = 6.0, 1.7, 1.7 Hz, 2H), 3.77 (s, 3H), 3.93 (s, 3H), 4.83 (br s, 1H), 5.08 (dt, *J* = 10.0, 1.7 Hz, 1H), 5.09 (dt, *J* = 17.0, 1.7 Hz, 1H), 5.92 (s, 2H), 5.99 (ddt, *J* = 17.0, 10.0, 6.0, 1H). ^{13}C NMR (125 MHz, $CDCl_3$): δ 28.1, 60.5, 61.7, 101.5, 114.3, 115.3, 131.7, 132.0, 132.8, 136.8, 137.2, 145.0. IR (neat): 3302, 2939,

1462, 1435, 1392, 1253, 1068, 1037, 910 cm^{-1}. EIMS (*m*/*z*): 238 (M^+). HRMS–ESI (*m*/*z*): [M + Na]$^+$ calcd for $C_{12}H_{14}NaO_5$, 261.0733; found, 261.0733. Anal. Calcd. for $C_{12}H_{14}O_5$: C, 60.50; H, 5.92. Found: C, 60.49; H, 5.87.

References

1. Taskinen, E. Thermodynamic, spectroscopic, and density functional theory studies of allyl aryl and prop-1-enyl aryl ethers. Part 1. Thermodynamic data of isomerization. *J. Chem. Soc. Perkin Trans.* 2. **2001**, 1824–1834, doi:10.1039/B101837J.
2. Chittimalla, S.K.; Kuppusamy, R.; Bandi, C. A detour route for meta functionalization of phenols. *Synlett* **2014**, 25, 1991–1996.
3. Loriot, M.; Robin, J.P.; Brown, E. Syntheses totales et etudes de lignanes biologiquement actifs—6: Syntheses totales de la (±)-iso-*β*-peltatine et de ses analogues. *Tetrahedron* **1984**, *40*, 2529–2535.
4. Coppola, G.M.; Schuster, H.F. The chemistry of 2H-3,1-benzoxazine-2,4(1H)-dione (isatoic anhydride). 21. A mild processfor the preparation of 10-alkyl-9-acridanones and it's application to the synthesis of acridone alkaloids. *J. Heterocycl. Chem.* **1989**, 26, 957–964.
5. McKittrick, B.A.; Stevenson, R. Synthesis of the yeast antioxidant benzofuran and analogues. *J. Chem. Soc. Perkin Trans.* 1 **1984**, 709–712, doi:10.1039/P19840000709.
6. Adesomoju, A.A.; Davis, W.A.; Rajaraman, R.; Pelletier, J.C.; Cava, M.P. Total synthesis of leucoxylonine. *J. Org. Chem.* **1984**, 49, 3220–3222.
7. Vshyvenko, S.; Scattolon, J.; Hudlicky, T.; Romero, A.E.; Kornienko, A.; Ma, D.; Tuffley, I.; Pandey, S. Unnatural C-1 homologues of pancratistatin—Synthesis and promising biological activities. *Can. J. Chem.* **2012**, 90, 932–943.
8. Lin, J.; Zhang, W.; Jiang, N.; Niu, Z.; Bao, K.; Zhang, L.; Liu, D.; Pan, C.; Yao, X. Total synthesis of bulbophylol-B. *J. Nat. Prod.* **2008**, 71, 1938–1941.
9. Dallacker, F. Derivatives of methylenedioxybenzene. XXVIII. Reactions of the dimethoxymethylenedioxybenzaldehydes. *Monats. Chem.* **1969**, 100, 742–747.
